# Evaluation of a national operational salmon lice monitoring system—From physics to fish

**DOI:** 10.1371/journal.pone.0201338

**Published:** 2018-07-31

**Authors:** Mari Skuggedal Myksvoll, Anne Dagrun Sandvik, Jon Albretsen, Lars Asplin, Ingrid Askeland Johnsen, Ørjan Karlsen, Nils Melsom Kristensen, Arne Melsom, Jofrid Skardhamar, Bjørn Ådlandsvik

**Affiliations:** 1 Institute of Marine Research, Bergen, Norway; 2 Norwegian Meteorological Institute, Oslo, Norway; Universitat Politècnica de València, SPAIN

## Abstract

The Norwegian government has decided that the aquaculture industry shall grow, provided that the growth is environmentally sustainable. Sustainability is scored based on the mortality of wild salmonids caused by the parasitic salmon lice. Salmon lice infestation pressure has traditionally been monitored through catching wild sea trout and Arctic char using nets or traps or by trawling after Atlantic salmon postsmolts. However, due to that the Norwegian mainland coastline is nearly 25 000 km, complementary methods that may be used in order to give complete results are needed. We have therefore developed an operational salmon lice model, which calculates the infestation pressure all along the coast in near real-time based on a hydrodynamical ocean model and a salmon lice particle tracking model. The hydrodynamic model generally shows a negative temperature bias and a positive salinity bias compared to observations. The modeled salmon lice dispersion correlates with measured lice on wild salmonids caught using traps or nets. This allows for using two complementary data sources in order to determine the infestation pressure of lice originating from fish farms on wild salmonids, and thereby provide an improved monitoring system for assessing risk and sustainability which forms the basis for knowledge-based advice to management authorities.

## Introduction

Norway is at present the world’s leading producer of Atlantic salmon and 1.24 million tons of salmon were sold from fish farms in 2016 (Statistics Norway). The value of this production was 59.9 billion NOK (7.19 billion USD). The Norwegian government has decided that the production shall continue to grow, provided that it can be done in an environmentally sustainable way [1]. This is thus implemented through the division of the Norwegian coast into 13 production zones. In each of these, an environmental indicator shall be used to determine if the effect is low, moderate or high. In these cases, the farming industry is allowed to grow (green), freeze production (yellow) or have to reduce the production (red). The system is therefore nicknamed the “Traffic light system” [2]. The only indicator used to measure sustainability is at present the effect of salmon lice (*Lepeophtheirus salmonis*), released from salmonid farms, on the mortality of wild salmonid fish [1].

The parasitic salmon lice feeds on mucus, skin, muscle and blood of their host, and infestations may scale from physiological effects to mortality [3]. The parasitic salmon lice are found only on salmonid hosts, in Norway this includes Atlantic salmon (*Salmo salar*), Sea trout (*Salmo trutta*), anadromous Arctic char (*Salvelinus alpinus*) and farmed rainbow trout (*Oncorhynchus mykiss*). The lice hatch directly from the paired eggstrings as pelagic nauplius I, which molts into pelagic nauplius II, and thereafter to the infective copepodids which remains pelagic until it attaches to a host. It takes approximately 40 degree-days from hatching to reach the copepodid stage. If unsuccessful in finding and attaching to a host, the copepodids die after 170 degree-days, leaving an infective window from 40 to 170 degree-days after hatching [4,5]. Later stages (chalimus I and II, preadult I and II, and adult) live on the host and are usually not infective. The stages that live on the host are divided into sessile (chalimus I and II) and mobile (preadult I, II and adult) lice based on their ability to move around on the fish. During the planktonic stages (nauplii and copepodid) the salmon louse is known to react to external stimuli as light and salinity by vertical swimming [6–9]. The lice react to light by migrating towards the surface during the day and sink down during night [9–10]. At the same time, it has been shown in laboratory experiments that salmon lice avoid low-salinity water [8,11]. Due to different results on the lower salinity threshold from the experiments, previous modeling experiments have used salinity at 20 as the lower limit [4,10,12]. And the low-salinity avoidance is assumed to be stronger than the light attraction due to physiological responses causing reduced survival in low-salinity water [11].

A continuous monitoring is needed to identify areas where the salmon lice densities are so high that that it poses an unacceptable high risk of mortality for wild salmonid fish. Several models describing the infection pressure from farms based on both hydrodynamic models [4–5,10,12–21] and statistical [2,22–25] models are developed. Helland et al. 2015 [26] unsuccessful attempting to predict infection on similar material as used here, using a statistical approach where infection pressure was estimated by distance. Their analysis showed a strong influence of environmental variables on infestation pressure, however, only monthly lice counts in farms was available, in addition to low resolution temperature data and using freshwater discharge as a proxy for salinity variations.

Over the last ~15 years models based on hydrodynamic circulation has been implemented as a tool to predict the drift of the planktonic stages of salmon lice with water currents [4–5,10,12–21]. These models have shown that the water currents may transport the lice tens of kilometers from the source farm [10]. Strong pulsating currents transport lice into the fjords, occurring about every 2 to 3 weeks, followed by a period of transportation out of the fjord again [10]. The pulsating nature of water currents are confirmed by current observations and often found to be more constrained to the surface and stronger during summer compared to winter. In addition to the calculated transport distances, the models have revealed lice densities with great spatial patchiness [27]. High density of lice is found to aggregate in smaller areas, often along land and within eddies. The high temporal and spatial variability of the current field make realistic salmon lice dispersion pattern difficult to calculate without knowledge of hydrodynamic circulation. The relatively long pelagic stage of the lice where the larvae drift with water currents means that the lice may infest a large area, and that the size of the infective area may be heavily affected by temperature as this affects longevity of the infectious stage [28–29].

The salmon lice particle tracking model has been developed to include realistic salmon lice release from all farms based on reported number of lice on farmed fish. The numerically calculated distribution of lice is shown to be in good agreement with the observed newly infested lice on wild fish [12], farmed fish [21] and fish in sentinel cages [4]. However, as discussed in Sandvik et al. 2016 [30], comparison of point measurements and model estimates in a fluctuating field is not trivial. In Sandvik et al. 2016 [4] salmon lice estimated with the particle tracking model was validated against salmon lice observations on fish in sentinel cages in the Hardangerfjord 2012–2016, using validation methods from numerical weather forecast [31].

Here, we present a mechanistic approach which includes both hydrodynamics and lice behavior. This kind of operational salmon lice model system facilitates a comprehensive approach including dynamical modeling of the lice infestation pressure, considering all environmental variables (circulation, temperature, salinity, wind) with local variations along the fjord axis, regional variations between neighboring fjords and national variations along the entire Norwegian coast. The development of salmon lice particle tracking models using hydrodynamic currents have roughly followed the same development in Norway as in Scottish and Faroese waters [15–20].

The establishment of an 800 m x 800 m horizontally resolved modelling system (NorKyst800) covering the whole Norwegian coastline, running operationally (real-time) by the Norwegian Meteorological Institute (MET Norway) has made operational salmon lice simulations with realistically hydrodynamical transport routes possible [32]. Based on results from NorKyst800, the Institute of Marine Research (IMR) runs the salmon lice model and publishes the results online every week, showing total number of copepodids summed over the last 10 days. The modeled distribution of copepodids is based on release of nauplii from all active fish farms. The number of nauplii is calculated from the reported level of adult females, biomass (number of fish) and temperature [24], provided by each farm on a weekly (temperature and adult females) or monthly (biomass) basis. The model results are used to guide the adaptive field monitoring part of the system, where regions with elevated salmon lice infestation pressure is confirmed by counting lice on wild trout.

The overall goal with this paper is to contribute to the knowledge base making the foundation of an environmental sustainable growth in Norwegian aquaculture. The specific objectives of this paper are (1) to describe the operational salmon lice model, and (2) to further assess the quality of the monitoring system, which are necessary for giving an accurate and robust assessment of the lice infestation pressure. For the first time, we can now present a hydrodynamic ocean model system that covers the entire Norwegian coastline and the quality of the modeled infestation pressure is tested against observations on wild caught trout and char. By using two complementary data sources; the operational model and wild fish data, we can provide an improved system for assessment of risk and sustainability, which forms the foundation for knowledge-based advice to management authorities.

In this paper, we will refer to the national operational monitoring system to both include the operational salmon lice model and the wild fish data, where the operational salmon lice model is a combination of a hydrodynamical ocean model and a salmon lice particle tracking model.

## Material and methods

### Background

The Atlantic salmon post-smolt leave their respective rivers during spring time and are exposed to salmon lice in the fjord and coastal region as they pass through on their way towards the open ocean. The post-smolt migration starts in the beginning of May in southern Norway, and the timing is delayed northwards, reaching the end of June in northern Norway [33–35]. The exposure time of the post-smolt to the lice in the fjord is also highly variable depending on the distance from the river to the coast and with the progressing speed and route of the smolt [34]. The sea trout and Arctic char spend more time in the coast or fjord during summer and is therefore susceptible to elevated infestation pressure for a longer time period, up to four months [36–37]. Field monitoring is focused on these two periods; the first period, which is most relevant for salmon post-smolt migration, and the second period which is most relevant for sea trout and arctic char, with several teams operating along the entire coast fishing for wild salmonids with trawls, traps and gillnets. The focus in this paper will be on the first period early in spring. This is done for two reasons; 1) The government has decided that the impact of salmon lice on wild salmon is the most important indicator controlling the aquaculture industry growth, thus far, and 2) The observational data is considered to be of higher quality in the first period. This because the infestation pressure normally increases during summer, the sea trout and sea char may show premature return to fresh and brackish water to mitigate lice infestations [38], thus both reducing the catchability of trout in fjords with elevated infestation pressure and complicating the interpretation of the observations in the second period. However, it is important to bear in mind that the impact of salmon lice on sea trout can be substantial, and often even stronger than the impact on salmon [39].

### Study area

**Fig 1:**
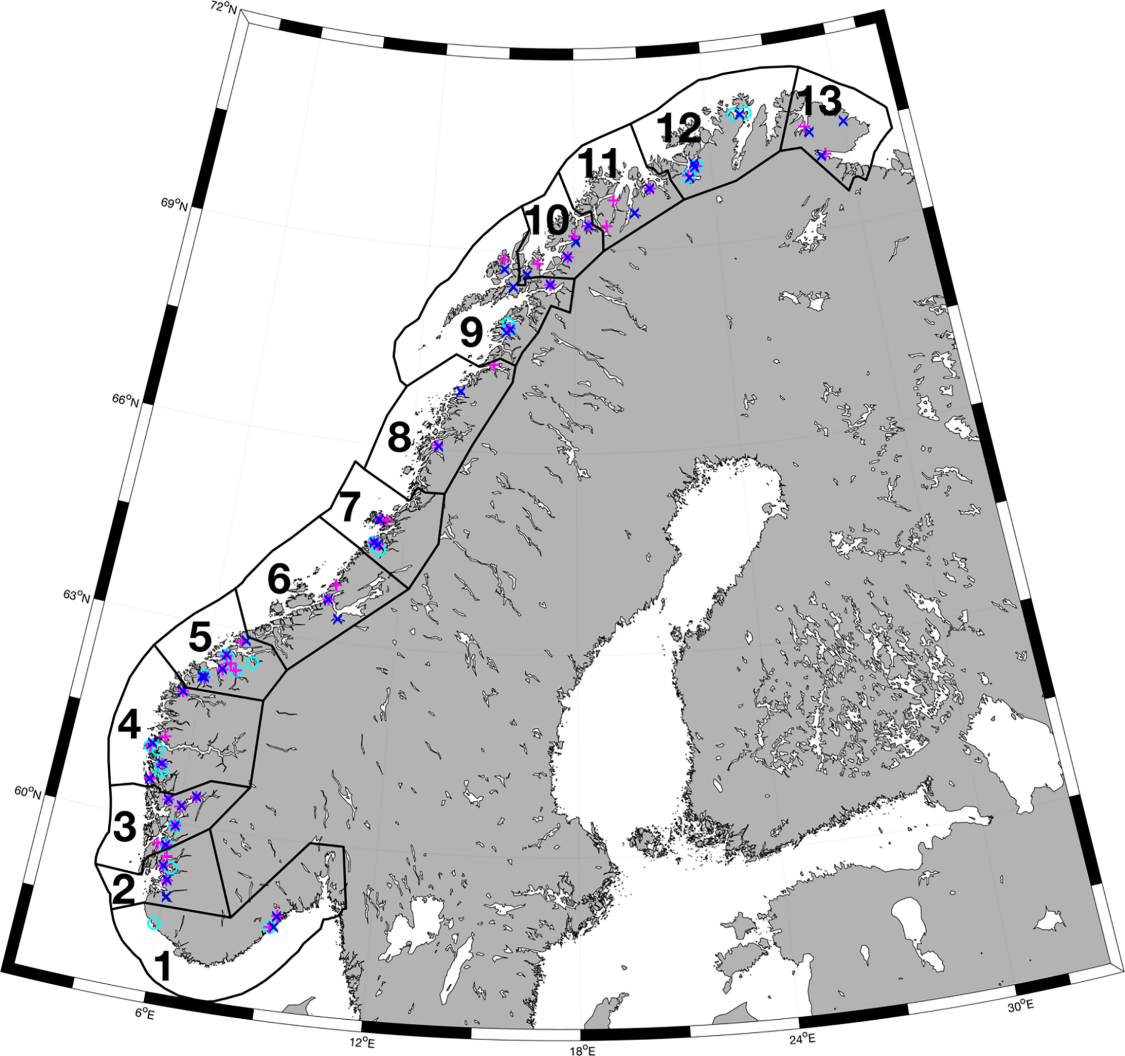
Map of study area. Stations where wild fish were caught during the national monitoring program for salmon lice on wild salmonids in 2015 (cyan), 2016 (magenta) and 2017 (blue), covering all the 13 production zones.

The study covers the entire Norwegian coastline, which is more than 100.000 km long including fjords, islands and bays and 25.000 km without the islands. A study area this large requires intensive effort and sophisticated monitoring tools, as morphology (coastline and bathymetry) is irregular and complex. The Norwegian coastal current provides a steady, though highly dynamic flow along the coastline, advecting salmon lice and other planktonic organisms northwards. The conditions in the fjords are strongly connected to the coastal water through baroclinic internal waves [10]. In the ‘production zone regulations’ (2017) the Norwegian coast was divided into 13 production zones (Fig 1). Ådlandsvik (2015) [40] used the NorKyst800 model to calculate the exchange of lice between all salmon farms in operation and clustered them together based on the connectivity. The clusters, i.e. the production zones, are constructed to have minimum connectivity between clusters.

### Hydrodynamic ocean model

The Norwegian Meteorological Institute (MET Norway) is responsible for operational ocean monitoring and forecasting of the Norwegian waters. The operational suite of ocean models at MET Norway utilize a one-way nested system consisting of three ROMS (Regional Ocean Modeling System, www.myroms.org) models [41–42]. ROMS is a free-surface, terrain-following, hydrostatic, primitive equations ocean model. The models are Arctic20 covering the whole Arctic Ocean using 20 km horizontal resolution, Nordic4 covering the North Sea, Nordic Seas and Barents Sea using 4 km horizontal resolution, and NorKyst800 as the innermost model covering the Norwegian coast with 800 m horizontal resolution.

The NorKyst800 model was developed through a collaboration between the Institute of Marine Research, MET Norway and The Norwegian Institute for Water Research (NIVA), and put in operational mode late in June 2012. Since then, daily averaged model results for the salinity, temperature, velocity fields and sea surface height have been publicly available, from http://thredds.met.no/thredds/catalog/fou-hi/norkyst800m/catalog.html.

The bathymetry and coastline in the NorKyst800 configuration were derived by interpolation of data from two high-resolution products (data with a resolution of about 50 m from Norge Digitalt [http://www.norgedigitalt.no] and the 900 m product from GEBCO_08 [General Bathymetric Chart of the Oceans, http://www.gebco.net]). Additional adjustments of the 800 m coastline were necessary to replicate the real coastline as well as possible and to open narrow straits less than 800 m wide but still important for the local circulation. To avoid model instability and/or spurious deep currents, the final masked bathymetry was smoothed.

The boundary condition formulations used in ROMS are the Chapman condition for free surface [43], Flather condition for 2D momentum [44], and nudging and radiation for tracers and 3D momentum [45]. There is also a 15 grid-point nudging zone encircling the domain where nudging towards the outer model is performed at a timescale of 50 days for tracers and 3D momentum. Subgrid-scale vertical mixing processes are parameterized using the Generic Length Scale (GLS) scheme, tuned to behave like the Mellor-Yamada 2.5 (k-kl) closure scheme using a parabolic wall function with free surface correction (k-kl_oc) [46–47], according to [48]. All models use 35 vertical levels, and parameters are chosen to get increased resolution toward the surface.

Atmospheric forcing is taken from the global ECMWF operational analyses and forecast (0.125-degree horizontal resolution) for the Arctic20 and Nordic4 models. For NorKyst800 we use the non-hydrostatic 2.5 km AROME MetCoOp regional atmospheric model [49], blended into the ECMWF fields [50] using a linear transition zone of 15 grid points to cover the parts of NorKyst800 that are outside the AROME MetCoOp domain. Fields of atmospheric forcing include mean sea level pressure, temperature at 2 m, u- and v-component of wind at 10 m, specific humidity at 2 m, rain rates and total cloud cover. Fluxes are calculated using ROMS internal bulk flux routine. The sea ice module in ROMS [51] is applied in order for the model to allow freezing of in-fjord surface water during winter.

Freshwater discharges for the Norwegian rivers are specified by daily climatological values based on historical results from the HBV model of the NVE (Norwegian Water Resources and Energy Directorate) [52]. Discharges are distributed linearly from the surface down to a prescribed depth for each river.

### Hydrographic observations

In order to assess the quality of the model results, we compared results for temperature and salinity during the period January 2015—December 2016 with observational data. For this purpose, relevant vessel data were retrieved from the Copernicus Marine Environment Monitoring Service (CMEMS, http://marine.copernicus.eu/), see S1 Fig. During this time period observations from vessels were taken at a total of 4732 positions inside the model domain (2015: 2601; 2016: 2131). These data were retrieved from CMEMS products INSITU_ARC_TS_REP_OBSERVATIONS_013_037 and INSITU_ARC_NRT_OBSERVATIONS_013_031.

Model results were retrieved from the dates and grid cells representing the time and positions of the observations. Then, observations and model results were integrated vertically using linear weights, in preselected *z* and *S* layers (*S* layers were defined as the vertical intervals between pre-selected isohalines in the observations). The linear weights were applied in order to account for irregular sampling in depth and salinity space. Representation in S layers were included since coastal systems are usually highly stratified in salinity. In the tables (Tables 1 and 2) that show evaluation statistics the results are computed over 5 depth ranges, as indicated in the leftmost column. Bias is defined as subtracting observations from corresponding model results. RMS is the root mean square difference between observations and their corresponding model results. For Table 2 the metrics were calculated between isohaline surfaces. Here, only observations from which temperature and salinity data are both available, are considered.

**Table 1:** Evaluation statistics including 2015 and 2016 over depth ranges.

*z* layer	Depth (m)	Temperature			Salinity		
		no.	Bias	RMS	no.	Bias	RMS
1	0–5	2637	-0.4	0.9	3342	0.94	1.44
2	5–25	2545	-0.2	0.7	2408	0.62	0.92
3	25–100	2468	-0.2	0.7	2328	0.34	0.54
4	100–300	1586	-0.6	1.1	1519	0.15	0.32
5	300–700	175	-1.0	2.1	174	0.01	0.17

**Table 2:** Evaluation statistics including 2015 and 2016 over isohaline surfaces.

S layer	Salinity range (obs)	no.	Temperature		Salinity	
			Bias	RMS	Bias	RMS
1	20.0–33.5	658	-0.3	0.9	1.84	2.30
2	33.5–34.5	1456	-0.1	1.1	0.64	0.81
3	34.5–34.9	1491	-0.1	1.1	0.29	0.50
4	34.9–36.0	1933	-0.4	0.7	0.11	0.26

### Salmon lice particle tracking model

The salmon lice model was developed with the purpose to map the horizontal distribution of infectious salmon lice from aquaculture sites in the water masses. The release of newly hatched lice larvae (nauplii) is calculated based on number of adult female lice reported from the aquaculture sites, as described by Stien et al. (2005) [24]. As the lice are hatched and released to drift freely in the ocean, the model calculates the transport along the currents as they undergo three planktonic larvae stages (two naupliar stages and one copepodid stage), where they are infectious to salmonid fish during the latter.

#### Model design

To calculate the transport of lice from a release position the lice dispersion model uses the horizontal current components where the transport of every particle is followed in a lagrangian field using 4th order Runge kutta scheme [53] with timestep (∂*t*) of 300 s. The position of every lice particle is stored every hour. The dispersion model uses reflective border at the surface. If the lice-particles were about to drifting onshore, they were held at its position until a shift in the currents transporting them away from the land border. Lice reaching the outer ocean border were removed from further calculations.

#### Age and mortality

The age of the lice particles was calculated in degree-days, being the integrated temperature over time. The lice were assumed to enter the infective copepodid stage at 40 degree-days [54]. Past 170 degree-days all lice were assumed to be dead from senescence and removed from calculations [54]. A constant mortality of 0.17 day^-1^ was assumed for all salmon lice, during all planktonic stages. This is a mortality on the scale that is normal for pelagic invertebrates of this size [6,55].

#### Vertical swimming

All salmon lice were given the ability to swim vertically. The swimming velocity was set to 5*10^−4^ ms^-1^ for all planktonic stages [56]. The direction was set to be upwards towards the surface when the light level exceeded a critical level of 2*10^−5^ μmol photon s^-1^m^-2^ during the nauplii stages and 0.392 μmol photon s^-1^m^-2^ during the copepodid stage [57]. When exposed to salinity levels under 20 the lice swam down [11]. When exposed to low salinity levels and light conditions, the low salinity avoidance was assumed to be the strongest trigger, and the lice swam down.

#### Light parameterization

The surface light (L0) at every salmon lice position is calculated from latitude (φ) and time of day (t), according to Skartveit and Olseth [57], where the level of irradiance was set to 1500 μmol photon s^-1^m^-2^ at midday and 5.76 μmol photon s^-1^m^-2^ at twilight, as described by Johnsen et al. [14]. From the surface to depth (z) the light is assumed to decrease according to Eq (1), where the attenuation coefficient (k) was set constant k = 0.2 [58]. The attenuation coefficient is known to correlate with salinity, oxygen and chlorophyll [58], but for the present investigation we found our simpler formulation to suffice [14].

$$Lz=Lo(\varphi ,t){\times e}^{-kz}$$ (1)

#### Diffusion

Due to lack of precise knowledge of subgrid scale processes and fluctuating swimming motion by the salmon louse itself, all lice were given a random movement component in both the horizontal and vertical direction [14]. The random component of the velocity (∂*Vr*) was given by Eq (2) where R is a random number normally distributed around 0, and *d* the diffusion constant is set to 0.02 m^2^s^-1^ horizontally and 10^−3^ m^2^s^-1^ in the vertical [59]. This is justified by the relatively high spatial and temporal resolution in the model forcing.

$$\partial Vr=R\sqrt{\frac{2d}{\partial t}}$$ (2)

#### Initialization and concentration calculation

The salmon lice model was run from 1^st^ of April to 31^st^ of August. As a part of the post-processing, the abundance of copepodids is calculated as lice concentration by aggregating the number of infective lice per grid cell (vertically integrated over the upper 20m) diurnally. When dividing by the grid area, the lice concentration is expressed as number of lice copepodids per m^2^ per day.

### Release of salmon lice from fish farms

The number of eggs hatched into the water is calculated from Stien et al. (2005) [24] based on weekly reported temperature at 3 m depth, number of female lice per fish and number of fish at each site, as described above. We assume that a female can produce 150 eggs per string [60]. In the model, the salmon lice nauplii are released from all active farm locations represented by 5 superparticles released every hour. The representation of salmon lice using superparticles was done to simulate the dispersion of a great number of particles and include the mortality in an efficient way where the computational power needed for the calculations is kept to a minimum. Each of the 5 superparticles represent 1/5 of the total nauplii number calculated for each release, regardless of the number nauplii released from the farm location. The maximum number of particles are 3 360 000.

Fish farmers report biomass and number of fish monthly to the Directorate of Fisheries (Fiskeridirektoratet). Temperature at 3 m and counts of salmon lice, in three classes: 1) sessile lice, 2) mobile lice and 3) adult female lice are reported weekly to The Norwegian Food Safety Authority (Mattilsynet). The regulations require that the lice must be counted on at least 20 fish every week (10 fish in the period June 1^st^ to January 31^st^), covering half of the cages [61]. These data are stored in a database at Norwegian Marine Data center at the Institute of Marine Research (IMR). A weekly summary of these data is provided to the model group and is used to calculate the source term, i.e. egg hatching, for the operational salmon lice modelling.

The number of fish in the farms is reported monthly to the authorities. Taking the median of the fish counts in successive months in 2015 gives a linear reduction of approximately 1% per month. These monthly data have been linearly interpolated to weekly values, to have the same time resolution as the other data set. After the last reporting from a farm, we assume slaughtering has taken place, and therefore let the number of fish decrease monotonically to zero for 4 weeks.

Temperature data at 3 m depth is reported together with the lice counts within Tuesday the week after the actual count. Most data fit into a band covering a reasonable seasonal cycle. There are however clear outliers, both warm and cold, and long stretches of constant temperature values are not realistic. Missing values are also a challenge. Since the nauplii production equation needs temperature input, the temperature series must be improved. There is for example uncertainty in the depth behavior of the salmon host and therefore the temperature experienced by the female adult lice. The objective is to provide reasonable temperatures at all locations throughout the year, using existing data. For each location and each week, the median of the 11 geographically closest reported values is computed, this value is used for the location that week. If there was a reported value for the location, it is replaced by this median giving a smoothing effect. The total number of 11 locations was chosen as a compromise between the desired robustness (filter out erroneous values) and locality.

### Observed infestation pressure on wild caught trout

The Institute of Marine Research (IMR) coordinates a national salmon lice monitoring program designed to monitor the infestations pressure on wild salmon and trout. Sampling is performed at several regular and irregular stations along the Norwegian coast using traps or gillnets [26,33], staying at each site for approximately two weeks. Several factors must be considered when deciding on the stations. Some are chosen for continuation of ongoing time series, some to ensure complete geographical coverage and some are chosen based on predicted elevated infestation pressure from the salmon lice model. The sampling is focused on two periods every year: May to June (during the smolt run) and June to August (during the sea-phase of the sea trout and char). Due to a strong temperature-driven seasonality in salmon lice infestation pressure an increase is expected from the first to the second observation period. However, in some years the catchability has been significantly reduced during the second round, especially in areas with elevated infestation pressure, and the quality of the data retrieved is considered to be lower. There might be several reasons for this, e.g. increased mortality and premature return to freshwater [39]. We will therefore only focus on the first period, which is most relevant for the salmon smolt migration, and do not include any data from the second period in this analysis. Due to logistical challenges the exact observation dates may vary between years and not all production zones are covered chronological from south to north. The exact timing of the collection of data from all the production zones is shown in S1 Table.

In the present analysis, we only included fish smaller than 150 g [62], based on the assumption that small fish migrate shorter distances [36,39,63] and therefore the observed infestation pressure would be a better representation of the local lice abundance near the location where they were caught. The data material is then comprised of 1088 fish from 21 stations in 2015, 1733 fish from 44 stations in 2016 and 2390 fish from 37 stations in 2017.

For describing the infestation pressure on wild caught fish, we calculated the mean abundance of copepodids, chalimus 1 and 2 on each station, and added these together into one group providing mean abundance of *young stages*. These young stages were assumed to better represent the local infestation pressure the fish had experienced just prior to being trapped, rather than the total number of lice which includes adults that could have been attached to the fish for months. The *relative intensity*, total number of lice per gram fish, was calculated on an individual level and averaged over all fish including both infected and non-infected, and named *averaged relative intensity*. The relative intensity is presented in categories: <0.1 lice/g, 0.1–0.2 lice/g, 0.2–0.3 lice/g, and >0.3 lice/g, as in Taranger et al. (2012) [62]. *Prevalence* was the number of infected fish divided by the total number of fish [64].

### The operational monitoring system

Throughout the year, several model simulations feed into the national operational monitoring system. The annual cycle of operations starts early in spring, due to the special focus on the salmon smolt migration. The output from the circulation model (nowcast) is supplied daily by MET Norway (running daily prognoses with warm start from yesterday’s model output) and downloaded to IMR. The salmon lice particle tracking model is operated by IMR and computations are performed every week with the latest sources (number of hatched eggs) calculated from the data that have been reported by the industry. To ensure realistic copepodid concentrations, the model is run for 40 days, where the first 30 days are the spin-up period and the results from the last 10 days are summarized and published online (www.lakselus.no). Personnel from IMR and associated partners from other research and management institutions assess the model output weekly and decide on locations with elevated infestation pressure that will be sampled to evaluate model predictions. An operational fishing team is then directed to the location with the objective to catch at least 50 fish per site. This procedure is continued through the summer. Both data sources, model and observations, are evaluated together and reported to the Norwegian Food Safety Authority several times a year.

When the operational season has passed, the model system is re-evaluated with updated data. The weekly reports of lice numbers and the monthly reports of number of fish from the farms are sometimes delayed, thus a rerun of the model system is necessary to ensure updated input data. Also, the hydrodynamic ocean model (NorKyst800) is rerun with updated forcing fields improving the quality of the hindcast compared to the nowcast. The updated model results provide the knowledge base for the annual risk assessment of the salmon lice infestation pressure on wild salmonids.

### Evaluation of the modeled infestation pressure

The model supplies copepodid densities hourly in the whole domain. When comparing this kind of model results with observations in a single point, the model output has to be processed and information about spatial variability is lost as the results are merged over several grid cells [4]. It is reasonable to assume that the fish caught in a specific location had been swimming around this location, within an unknown radius of the fishing location. We have therefore included many different averaging areas to investigate the sensitivity to averaging area. Horizontally the modeled density is averaged within boxes of different size, 1x1, 3x3, 5x5 and up to 29x29 grid points, centered around the observation point. A wide number of box sizes was included to investigate if the correlations would converge or reach a maximum. The output is then summed over three weeks, including the two weeks of sampling and one week prior to sampling. The timing is equal for all stations within a production area, and increasingly delayed progressing northwards (see S1 Table for more details). The week prior to sampling is added to account for lice that infested the fish prior to the sampling period. A summary of correlations between the extracted variables are found in Table 3. The correlations are calculated for 21 stations in 2015, 44 stations in 2016 and 37 stations in 2017. As described in [19] and [4], the observations of lice on fish are generally zero-inflated. Ordinary validation techniques used for homogenous physical properties (e.g. sea level, salinity, current velocity etc. [48]) are not suited for this kind of comparison, hence Spearman rank correlation was used in this case.

**Table 3:** Correlations between modeled and observed lice.

	2015		2016		2017		2015–2017	
	Young	Rel.int.	Young	Rel.int.	Young	Rel.int.	Young	Rel.int.
**1x1**	0.59	0.61	0.49	0.56	0.46	0.47	0.48	0.50
**3x3**	0.60	0.61	0.53	0.60	0.49	0.48	0.51	0.52
**5x5**	0.58	0.60	0.58	0.61	0.51	0.50	0.54	0.55
**7x7**	0.60	0.61	0.61	0.64	0.73	0.74	0.65	0.66
**9x9**	0.65	0.68	0.66	0.68	0.79	0.79	0.68	0.69
**11x11**	0.66	0.69	0.66	0.68	0.80	0.80	0.69	0.70
**13x13**	0.66	0.69	0.68	0.70	0.81	0.81	0.70	0.71
**15x15**	0.65	0.68	0.69	0.70	0.82	0.82	0.71	0.72
**17x17**	0.67	0.71	0.73	0.74	0.81	0.81	0.71	0.72
**19x19**	0.70	0.74	0.73	0.74	0.81	0.81	0.72	0.74
**21x21**	0.70	0.73	0.74	0.75	0.82	0.83	0.73	0.75
**23x23**	0.70	0.74	0.74	0.76	0.81	0.82	0.73	0.75
**25x25**	0.70	0.74	0.74	0.76	0.79	0.80	0.72	0.75
**27x27**	0.70	0.74	0.74	0.76	0.76	0.78	0.72	0.74
**29x29**	0.70	0.74	0.76	0.78	0.78	0.79	0.73	0.75

Spearman rank correlation between observations; mean abundance of young stages (Young) and averaged relative intensity (Rel.int.), and modeled lice densities within; 1x1, 3x3, 5x5 and up to 29x29 grid points for 2015, 2016 and 2017 individually and the whole time series combined.

## Results

### Evaluation of the hydrodynamical ocean model

In order to assess the quality of the model results, we compared modelled temperature and salinity from January 2015 to December 2016 with observational data. The results are given in Table 1, in selected *z* levels. Generally, there were a negative bias in temperature and a positive bias in salinity in the model results, meaning that the model generally simulates colder and more saline water than was observed. The salinity bias was particularly strong near the surface. To examine this shortcoming in more detail, the analysis was repeated with *z* layers replaced by *S* layers, i.e. the region between isohaline surfaces in the observations (Table 2). Here, we include only observations where both temperature and salinity data are available. The results are presented in Table 2.

The challenge with a strong positive salinity bias is more pronounced where low salinities are observed. The misrepresentation when salinity is >34.5 is much more modest. We also take notice of the fact that the cold bias is very low in waters with salinity in the range [33.5–34.9], but with a relatively large root mean square difference.

**Fig 2:**
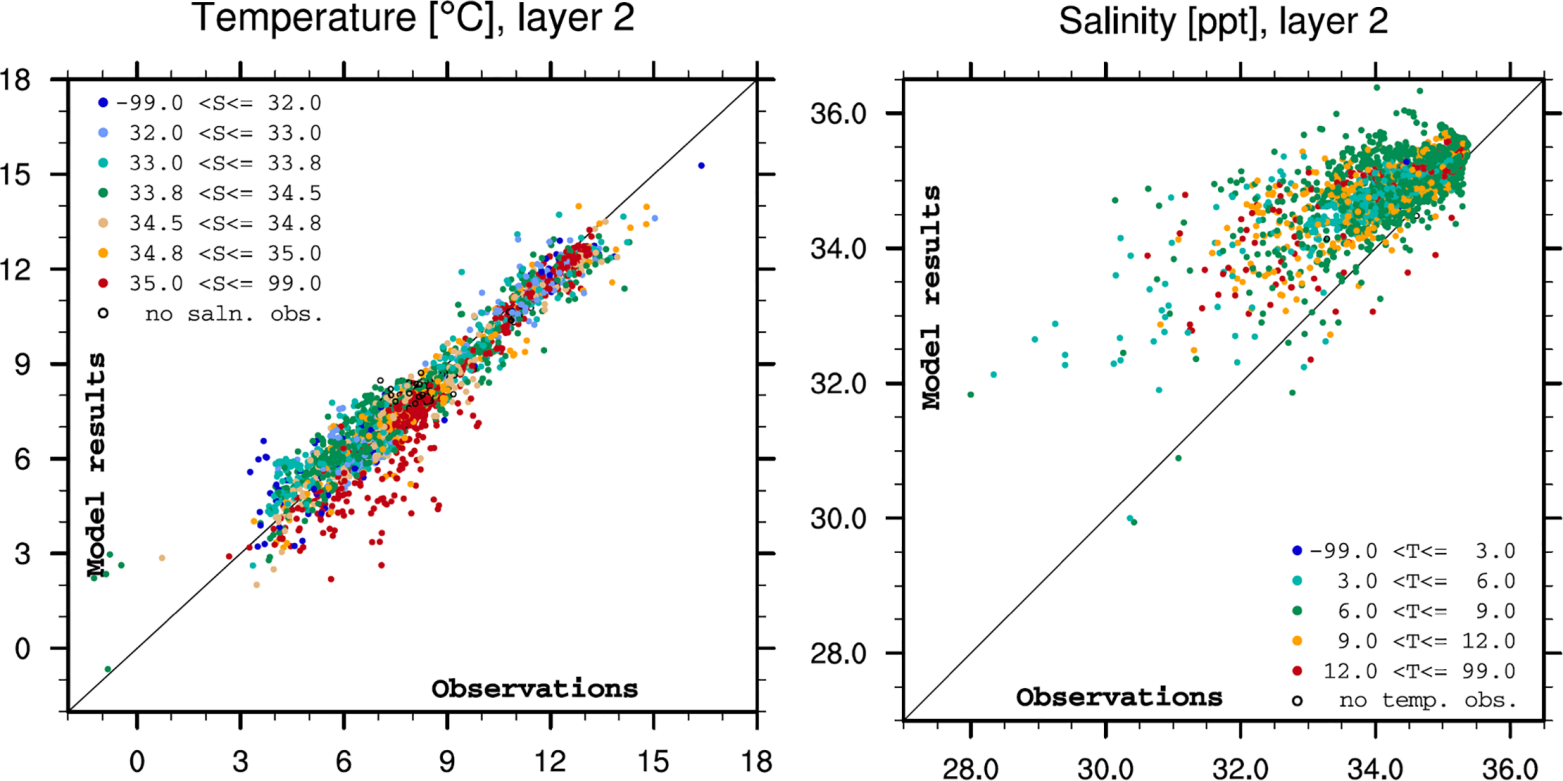
Hydrographic comparison. Scatter diagrams for temperature (left panel) and salinity (right panel) from the observations in layer 2 in Table 1. Observations and model values are given as the horizontal and vertical axes, respectively. The color coding corresponds to the observed value of the alternative variable, i.e. salinity in the left panel and temperature in the right panel. The color coding is shown by the inset graphic legends.

In Fig 2 we examine evaluation results in physical variable space as shown by the scatter diagram for *z* layer 2. First, note that the color coding reflects the observed value for the alternative variable, which provides information about the water type represented by each observation. For temperature (left panel) we find that the cold bias reported for *z* layer 2 (Table 1) is primarily a result of too cold conditions in relatively cold waters with high salinities. A corresponding analysis for *z* layer 1 (not shown) reveals that the cold bias is enhanced in the upper 5 m due to negative temperature offsets in warm, low salinity water masses.

With regard to the results for salinity, displayed in the right panel of Fig 2, the positive salinity bias reported above is again obvious. We note that salinity values above 35.4 have not been observed in the 5–25 m *z* layer, while the model results include higher salinities than this value. Moreover, the model has a challenge when it comes to reproducing the lowest salinities, making the range of salinity results significantly smaller in the model results than in the corresponding observations.

In an attempt to ameliorate the discrepancies in results for salinity, nudging of the 3D salinity to the model configuration which provides boundary conditions were introduced in the nested model system. The results displayed in S2 Fig suggest that the nudging has improved the results in *z* layer 2 and in the layers below. In the upper 5 m (*z* layer 1), most of the improvement took place prior to late 2015, but the nudging introduced at that time may have had a stabilizing effect on the quality of the salinity results from NorKyst800.

### Near real-time distribution of salmon lice and evaluating the modeled infestation pressure

**Fig 3:**
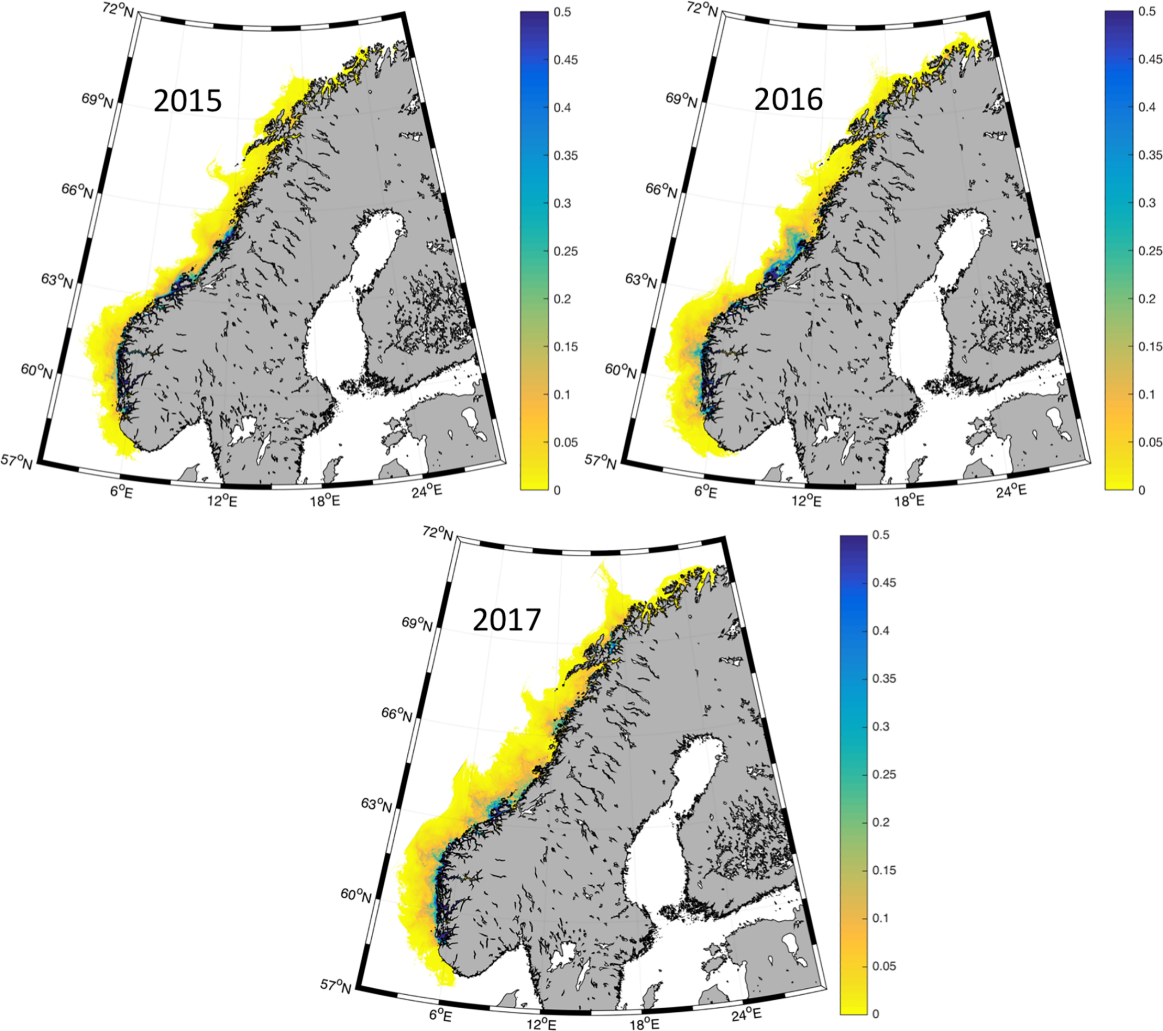
National distribution of salmon lice. Integrated lice density (#cop/m2) during May in 2015, 2016 and 2017.

Maps of the distribution of salmon lice are published weekly online starting in beginning of April and continuing through the summer season. Fig 3 shows accumulated salmon lice abundance during May from the hindcast, comparing 2015, 2016 and 2017. Salmon lice are abundant from the southern tip to the northern cape of Norway, with several hot-spots at the west coast and in the central part. The level is comparable between the years, but with high spatial variability. Many regions have local fallowing regimes which strongly affects the interannual variations in regional infestations pressure. In addition, there is a south-north gradient in lice densities due to both high numbers of fish farms in the south and a temperature effect on the lice reproduction rate. The model results indicate higher infestation pressure in 2016 and 2017 compared to 2015, and the highest infestation pressure was found in middle of Norway (zone 6 and 7, ref Fig 1) in 2016. The lice distribution did not extend as far northeast in 2015 as it did in 2016 and 2017. The overall distribution extended further offshore during 2017, especially from the middle part and northwards.

To evaluate the quality of the modeled lice density from the hindcast, we compared the results with observed lice infestation pressure on the wild caught trout and char through mean abundance of *young stages* and *averaged relative intensity*. The terminology and method for comparison was explained in the method section and the results are presented here in Table 3. The correlation coefficient varied between 0.46 and 0.83, where all are significantly different from zero. The correlation was generally higher when including larger model areas, and higher when using averaged relative intensity from the observations. There were only minor differences between 2015 and 2016, however 2017 started out with lower correlations at small areas compared to the two other years and then ended up with higher correlations for the large areas. When the three years were merge, the Spearman rank correlation still stayed above 0.7 for domains larger than 13x13. The analysis was done for both separate years and the whole series combined to investigate if there were systematic differences between the years, that would be masked if only the combined series were provided. The correlations started flattening out at grid size 9x9 and the first top was reached at 11x11 (for averaged relative intensity in 2015), while the other variables reached maximum values at larger grid sizes and some converged towards a constant value.

**Fig 4:**
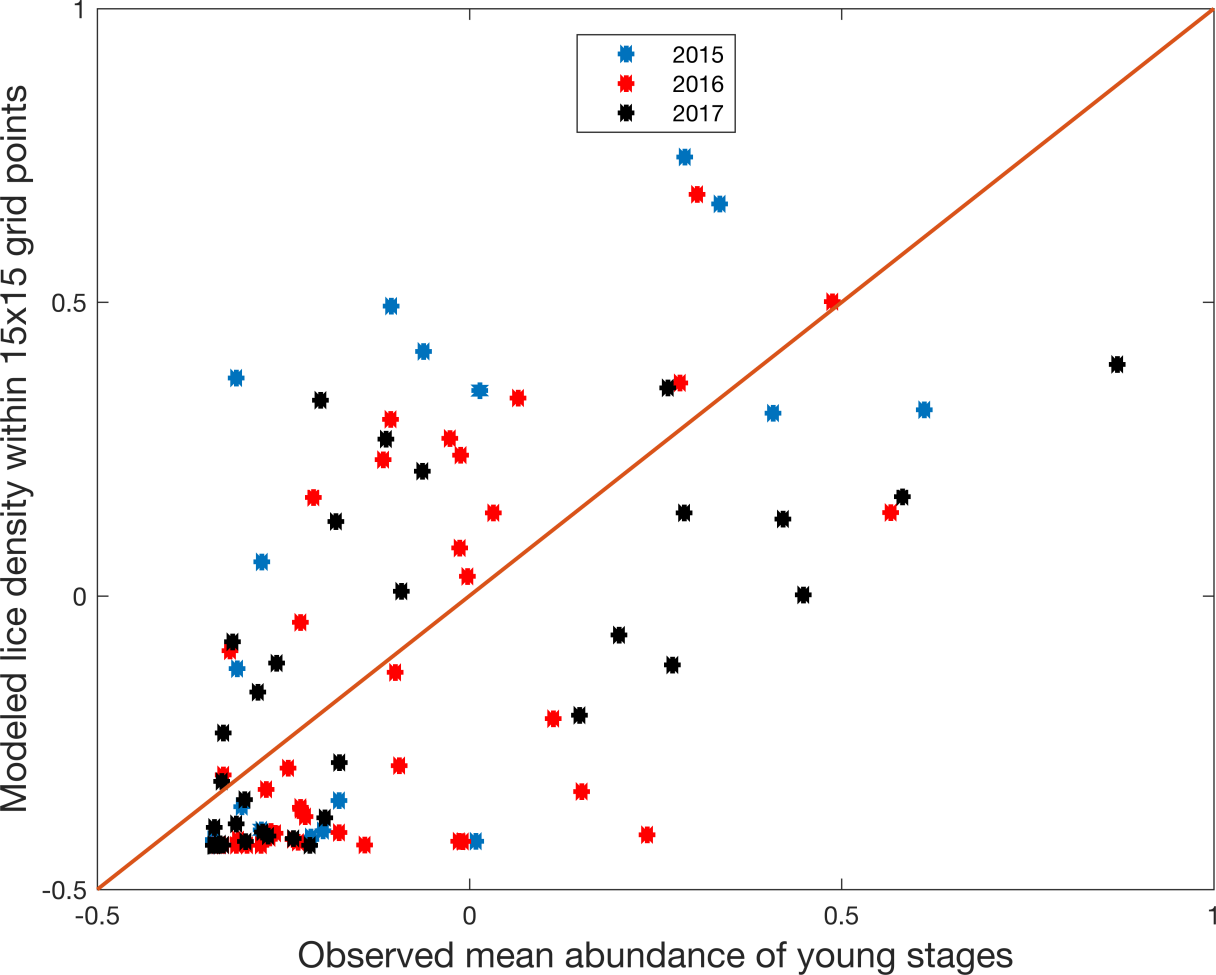
Comparing modeled and observed lice. Observed mean abundance of young stages plotted against modeled lice density within 15x15 grid points of the observations point for 2015 (blue), 2016 (red) and 2017 (black). Both variables are standardized for comparison (subtracted the mean and divided by standard deviation) and plotted on a log-log scale.

Fig 4 shows the comparison between observed mean abundance of young stages and modeled number of copepodids from the hindcast within 15x15 grid points for all stations including both 2015 (n = 21), 2016 (n = 44) and 2017 (n = 37). The grid size 15x15 was chosen for illustration because this was the size where the strongest correlation curve (young stages in 2017) reached the first top, however the pattern shown here was very similar across all sizes above 9x9. There were no systematic differences between the three years, however low densities of lice were observed more frequently than high densities.

### Model complementing observed lice infestations

**Fig 5:**
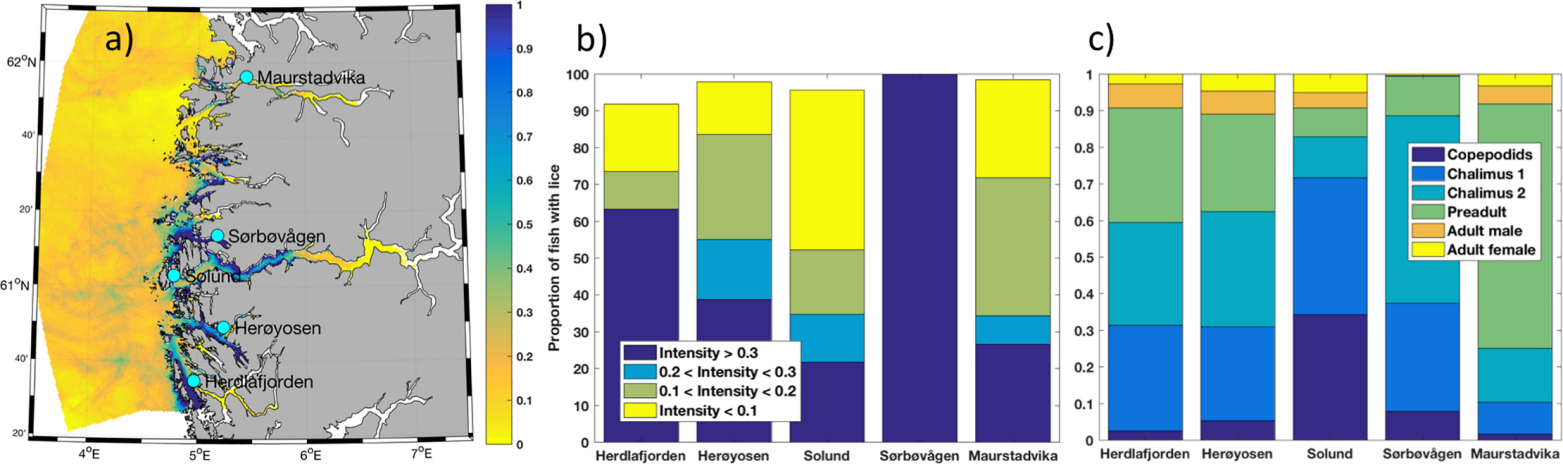
Production zone 4. Modeled lice density from May 23^rd^ to June 12^th^ 2016 (a) in production zone 4 showing the position of five locations with wild fish data. Relative intensity (b) divided into categories; <0.1 lice/g, 0.1–0.2 lice/g, 0.2–0.3 lice/g and >0.3 lice/g, and distribution of lice stages (c) for the five locations; Herdlafjorden, Herøyosen, Solund, Sørbøvågen and Maurstadvika.

Observed infestation pressure on wild caught trout provide valuable data for model comparisons, but when assessing the overall risk and sustainability within a production zone discrete observation points are not sufficient to evaluate the overall infestation pressure in a region. The operational salmon lice model is continuous in both time and space, and by using these two complementary data sources together the assessment is improved. Two examples are shown here, where the model results provide a more complete description of the infestation pressure in 2016 from production zone 4 and 10 (ref. Fig 1). In production zone 4, the lice density was high in the southern part while lower in the northern part except for some local hotspots (Fig 5A). Capture of wild trout was done on five locations in this area; Herdlafjorden (n = 49, number of fish), Herøyosen (n = 49), Solund (n = 23), Sørbøvågen (n = 9) and Maurstadvika (n = 64), covering the whole geographical range from south to north. The number of samples from each site was corrected for size (<150gr), and the average weight of the remaining fish was 51.1 grams. Almost all the fish from these locations had experienced a considerable infestation pressure with prevalence above 92% on all stations, and many (ranging from 22% to 100%) had a relative intensity above 0.3 lice/gr (Fig 5B). The salmon lice stage distribution varied significantly between the stations ranging from 25% to 89% young stages (Fig 5C).

**Fig 6:**
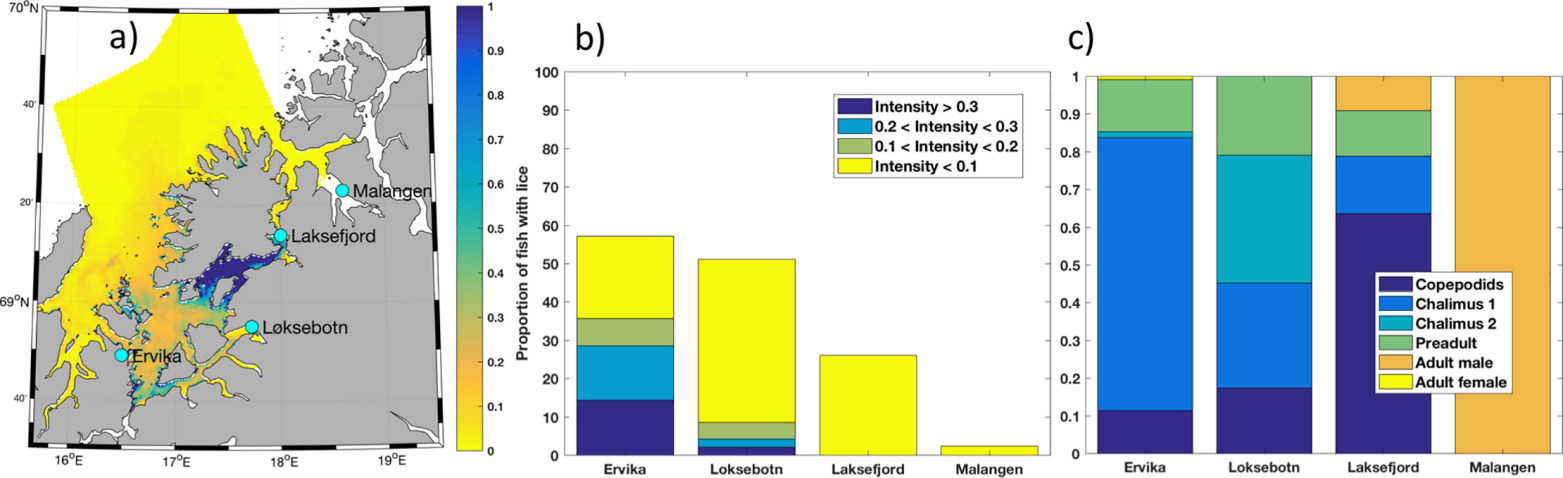
Production zone 10. Modeled lice density from June 20^th^ to July 10^th^ 2016 (a) in production zone 10 showing the position of four locations with wild fish data. Relative intensity (b) divided into categories (see Fig 5) and distribution of lice stages (c) for the four locations; Ervika, Løksebotn, Laksefjord and Malangen.

Most of production zone 10 had low lice density while an area of high densities was found in the strait between the island Senja and the mainland (Fig 6A). Thus, this was an example where all of the observations are from locations with low infestation pressure, while the model showed an area with higher levels in between. Trout were caught at four stations in this area; Ervika (n = 14), Løksebotn (n = 47), Laksefjord (n = 50) and Malangen (n = 42), with average weight of 66.9 grams for fish below 150 grams. Lice were found at all stations but with low to medium prevalence, 2%-57% (Fig 6B). Fish with relative intensity of 0.3 lice/gr or higher were only found on two stations, Ervika (14%) and Løksebotn (2%).

## Discussion

### Evaluation of the hydrodynamical ocean model

When comparing the model with observations, there were minor differences in temperature, less than 0.4 in the upper 100 m, and moderate differences on salinity, less than 0.94 in the upper 100 m. The time evolution of monthly temperature and salinity biases in the operational NorKyst800 model since its initialization late in June 2012 is shown in S2 Fig. During this 4.5-year period, the only major modification in the operational configuration took place in November 2015.

The excessive positive salinity bias values seen in the right panel of S2 Fig prompted an investigation with a focus on the role of open boundary conditions for the evolution of salinity in NorKyst800 configurations [65]. Melsom and Trodahl (2016) [65] noted that the model which provides conditions along the open boundaries of NorKyst800 in the operational configuration have higher salinities in the upper layers than two alternative basin-scale models. Next, they ran a set of three 1-year experiments for the NorKyst800 domain that differed only in the provision of open boundary conditions. Their analysis revealed that both of the alternatives to the present operational configurations performed better when compared to salinity observations from the final 6-month period. The study by Melsom and Trodahl (2016) [65] thus concluded with a recommendation that the outer model which provides conditions at the open boundaries of the NorKyst800 domain should be replaced when the operational configuration is revised. In order to properly account for variability, nesting with an outer (basin scale) model which assimilates observations is the configuration of preference.

The modeled salinity bias is largest in low salinity water, and therefore will this discrepancy be most dominant in the inner fjords with large river runoff. Since the model is running in an operational mode, real-time runoff data is not available and climatological values are used. Updated river runoff is available when the model system is revisited during fall, which improves the quality of the hindcast compared to the nowcast. The salmon lice are known to avoid low salinity waters (<20) by vertical migration [11], this avoidance mechanism may be underestimated in the operational model. The lowest salinities are found in the innermost fjords, in these regions the model will provide too high abundance of lice near the surface. In addition, this will affect the horizontal distribution since lice situated near the surface are more likely to be transported out of the fjord while lice deeper in the water column are transport into the fjord [14]. At the same time, a model with 800 m horizontal resolution is too coarse to resolve dynamics in narrow sounds and small fjords. Higher resolution, e.g. 160 m, is necessary when studying smaller systems and local processes [10,21]. The 800 m model is well suited for studying regional processes while local processes within small fjords should be assessed with care. However, with the computer resources currently available, this is the highest resolution possible for full operational coverage of the entire coast.

### Evaluation of the modeled infestation pressure

Helland et al. 2015 [26] highlighted statistical and ecological challenges regarding monitoring of lice infestations on wild salmonids. Specifically, the observations of total lice on fish are zero-inflated, which means high number of observations with low or zero abundance and a few observations of high abundance of lice. This is a common distribution of parasites on hosts. However, as the farming intensity has increased and the release of lice has increased accordingly, the number of low abundance observations has been reduced in the recent data set (2015–2017) compared to the analyses based on data from 2004 to 2010 made by Helland et al. 2015 [26]. In addition, the sampling procedure has changed. Previously the fish was only caught with gillnets, frozen and transported to the lab before counting the lice, while now the fish is mainly caught with traps and the lice is counted in the field shortly after catch by trained personnel. The zero-inflated distribution of observed lice infestations is clearly seen in Fig 4. The accuracy of the modelled estimate is variable in regions with low infestation pressure. While the observations display a wide range of low values, the model has a very narrow range of low estimates. In areas with low infestation pressure the comparison is generally more sensitive to temporal and spatial mismatch, in addition the contribution of lice from wild fish is a potential source which is not included in the model.

We have chosen to focus only on fish <150 g, and the young lice stages. The size is chosen as these represent fish that has left the rivers for the first time and is less likely to have stayed in the fjords during winter [39]. In addition, the habitat of these is restricted to a few kilometers from their river of origin and therefore may better represent the local infestation pressure. Also, as premature return to the river is observed as a response to heavy lice infestation pressure [38], observations later in the season and on larger fish becomes more complicated. Previous analysis has indicated that temperature is a strong driver of the infestation pressure [26,33,66], due to both that temperature in this period increases with season and due to a faster reproduction rate with increasing temperatures. Helland et al. 2015 [26] also found a strong correlation with fish size, which may either be due to that larger fish have stayed longer in the sea or that small fish often have more sessile lice that is easier lost during freezing and thawing.

The objective of using statistical modeling [26,33,66] is to find which variable(s) that can best explain the variation of the observations at hand. It is important to keep in mind that mechanistic modeling as described in this monitoring system, is the complete opposite approach, where the objective is to include all available information regarding the processes relevant to the organism in focus. By modeling causal links between the environment and dispersal of lice, the operational system can provide a realistic infestation pressure along the entire Norwegian coast. The model is not depending on input from the sparse observations of lice on wild salmonids to provide results, however the comparison between the two, as shown here, shows that the model system is suited for the purpose. The strength of the monitoring system, including both observations and modeling, is that these two independent data sources are complementary and in total will give a much better description of the actual conditions.

### Model complementing observed lice infestations

The main objective for developing a national operational monitoring system, including both an operational salmon lice model and the wild fish data, is to provide an improved system for assessment of the risk and sustainability in all production zones. Figs 5 and 6 are included to illustrate the importance of both data sources. If the assessment of production zones 4 and 10 only were made based on the wild fish data (b), zone 4 would be classified as a high risk area while zone 10 would be a low/moderate risk area. However, when including the modeled infestation pressure (a) into the assessment of zone 4, it is clear that the area of high infestation pressure does not cover the entire production zone and the spatial variability within this region is very high. Considering zone 10, the modeled infestation pressure shows that an area of high infestation pressure was not covered by the wild fish data, which means that the overall risk in the area was higher than the observations showed. It is therefore very important to consider these two data sources as complementary, since a single source might under- or overestimation the risk.

### Sources of uncertainty

The spatiotemporal distribution of salmon lice is strongly affected by the source term, i.e. the release of salmon lice from fish farms. High quality of the reported data is essential and more exact information about counting time could improve the reports [4]. If the fish farmers report late or not at all, the operational model system cannot accurately calculate the infestation pressure in a region. However, the rerun during fall includes also delayed reports and interpolates the values to fill the gaps. The difference between the two simulations is shown in S3 Fig, for production zone 10 as was previously discussed. The figure from the operational salmon lice model a) shows copepodid density accumulated over 10 days centered around 2^nd^ July 2016, and included all the information available at that time and formed the bases for making a decision on fishing locations while b) is identical to Fig 6B. The locations in this region were mainly chosen to represent important areas for salmonid fish in the area, only including areas with moderate infestation pressure. The difference between a) and b) is considerable, where the direct operational product shows a clear underestimate of the infestation pressure. The difference is mainly due to late/missing reports from fish farms in the area (www.BarentsWatch.no) in the first approach, which are included in the second approach. It is therefore important to emphasize that the operational product (nowcast) mainly functions as an early warning system identifying high risk areas, while the rerun (hindcast) is used for developing knowledge-based advice to management authorities.

A challenge with the use of lice infestation pressure derived from wild caught fish, is that the history of the individual fish is unknown, i.e. where the fish has collected the observed lice. The location of the fishing gear is probably not a representative position for exactly where most of the fish in a specific sample have resided the last few weeks. Sea trout is known to have a stationary behavior in inner fjord systems [63], however they can occasionally migrate over longer distances (>500 km) [37]. To account for these challenges, we used several averaging areas from the model (1x1, 3x3, 5x5 and up to 29x29 grid cells) to investigate the sensitivity to resident area. The grid size 15x15, where the strongest correlation curve reached the first top, is used for illustration in Fig 4. This box covers a distance of 12 km and an area of 144 km^2^. Tagging studies of sea trout in western Norway recaptured most fish within 10–15 km from the released river, while all were captured within 70 km [37,67]. These distances are comparable, however the individual differences between single fish could be considerable. The difference among the averaging areas were not large, although an increase in correlation with increasing area was seen before they flattened out. The sensitivity to averaging area seems to be low.

The observations of salmon lice infestations on sea trout is used in the annual risk assessment of the environmental impact of Norwegian salmon farming [62]. However, recent investigations comparing trawled trout and salmon have shown that there is a correlation between lice levels, but also that trout generally has higher abundance of lice than salmon [68]. These results are important to consider when assessing the overall risk in a production zone but will not affect the correlation between modeled infestation pressure and observed lice on wild fish found here.

Even though the model is used to choose observation locations, the model and the observations are still independent of each other. However, the distribution of observations is not independent. A regular grid covering the entire Norwegian coast would have had a more skewed distribution towards low infestation pressure. The quality of a monitoring system should not be evaluated only on its ability to predict low risk areas but mainly on its ability to predict high risk areas. We therefore consider it to be an advantage that the dataset contains a higher portion of observations from areas with elevated infestation pressure.

Direct measurements of infective copepodids in the pelagic phase are lacking [16], and consequently we have to compare the modeled lice density with number of attached lice on fish. This approach adds more uncertainty related to infection efficiency (depending on temperature, salinity and age [69]) and mortality of lice between attached stages on the fish. Previously this model system has been validated against infestation levels in sentinel cages [4], however observations indicate that sentinel cages underestimate the infection level compared to wild fish due to the feeding behavior of wild sea trout [70]. The comparison performed in the present paper is the first approach using the whole national dataset on wild caught fish for model evaluation. The results are promising and provides confidence in the management system which is developed, and initiates further evaluation work as more data becomes available and the observations program is continued.

## Conclusions

### Major findings

The operational salmon lice model, including both the hydrodynamic ocean model and the salmon lice particle tracking model, has been running for several years and has proven to be reliable in the production of salmon lice infestation pressure in near real-time all along the Norwegian coast. With regard to quality, this paper has showed good comparisons between model and observations for both hydrographic properties (Fig 2) and salmon lice dispersion (Fig 4). Previously the salmon lice model has been validated against sentinel cages in the Hardangerfjord [4], while here we have shown that the model is valid along the entire coast. Updated infestation pressure all along the coast is invaluable information for evaluation of the carrying capacity of wild fish with respect to salmon lice, and hence being an important measure for the management of the salmon industry towards sustainable growth. This model system will, in combination with other model systems (e.g. [25]) and field observations, be included in the knowledge base which is needed for implementation of the new “traffic light” management system [1,2].

The national monitoring program for salmon lice on wild salmonids in Norway aims at describing the infestation pressure on wild fish in all areas with intensive salmonid farming. This is a substantial task, indicating the need for an adaptive surveillance based on predictions of areas with high lice infestations on wild fish, which may be verified by subsampling of wild salmonids. Implementation of an operational salmon lice model makes it possible to monitor the infestation pressure along the entire Norwegian coast in near real-time. Here we have demonstrated that a fully mechanistic approach covering the entire Norwegian coast is feasible both technically and qualitatively. The maintenance of a fully operational model is technically challenging and has been made possible through the cooperation between IMR and MET Norway.

Since the model is able to predict areas of high infestation pressure, and by extensive use of an adaptive monitoring where areas with high infestation pressure is verified using subsampling, the locations where wild fish are captured are not randomly selected. This will affect the overall assessment for an area if the observations of salmon lice on wild fish are interpreted by themselves. In addition, field monitoring is time consuming and expensive especially in a country like Norway where the coast is long, complex and many locations are remote. Therefore, in order to evaluate the infestation pressure in the 13 production areas, observations and model results must be considered complementary and should always be used together. Here we have shown that the quality of the model results is good at the monitoring stations, with a Spearman rank correlation above 0.7 for domains larger than 13x13, and the model is thus well suited for evaluation of the infestation pressure also between the monitoring stations. By combining these two data sources, it is possible to assess the overall infestation pressure in a production zone. Field monitoring will continue, and comparisons are done continuously to make sure that the quality is maintained also in the future.

### Value for management

Implementation of a national operational monitoring system makes it possible for the first time to assess the infestation pressure from salmon lice along the entire Norwegian coast in near real-time. This allows for a quick identification of high risk areas where salmon and trout will be especially vulnerable for salmon lice infections. It is important to identify salmon lice “hot spots” within the production zones and document with observations the salmon lice intensity in these areas. It is also of crucial importance to have the possibility to monitor the extent of a high risk area within the production zone and the time evolution of this fraction. Altogether this makes it possible for the government to initiate necessary measures to mitigate the high infestation pressure when it becomes a problem for wild salmonid fish. According to the salmon lice risk index that estimates increased mortality due to salmon lice infections made by Taranger et al. 2015 [62], all infections above 0.3 lice/gr are assumed to cause 100% mortality. If the total lice-induced mortality within a production zone exceeds 30% on a population level, the salmon production has to be reduced as a part of the ‘Traffic light system’ [1,2].

We have proven the quality of a modeled infestation pressure on wild fish, far away from the source namely fish farms. It is therefore reasonable to assume that the model resolves the most important transport mechanisms of salmon lice, specifically ocean circulation [10] and lice behavior [21]. This is important since the government has decided that salmon lice induced mortality on salmon smolt during migration from the river to the open ocean, will be the index for controlling sustainable growth. The model system we have described here is well suited for providing the infestation pressure that can be used for calculating number of lice on salmon smolt migrating through a fjord system.

The operational model system is also suitable for developing a lice mitigation strategy for fish farmers. The system can determine how the treatment, including different timing, at the various farms in a production zone affects the regional infestation pressure. Next, it is possible to test various model scenarios where different positioning and size of farms can be evaluated, in addition to increase and decrease in production.

## Supporting information

S1 Table Timing of the observations. Two-week intervals of fishing in the different production zones, added one week ahead of the fishing period for extraction from model.
(DOCX)

S1 Fig Distribution of available observations from cruises during the period 2015–01–2016–12. Each observation was assigned to the nearest grid cell in the model domain. The sizes of the dots are scaled so that their areas correspond to the number of observations in each grid cell. The dot size in the legend corresponds to the area when one observation is available. The magnitude of the model biases is given by the color coding which is defined in the graphic legend below the panels. The 1000 m depth isobaths are indicated by a black line.
(TIF)

S2 Fig **Time series of differences between model results and observations for temperature (left) and salinity (right).** Differences are positive when model values are higher than the corresponding observations. Displayed here are monthly means of the layer average differences at the positions from which observations are available. Results are displayed for the three selected z layers as indicated by the line legends, corresponding to layers 1, 2 and 4 in Table 1. Thin lines bridge months with no data.
(TIF)

S3 Fig Comparing nowcast and hindcast. a) Operational model system, map used to select fishing location, b) Rerun of model system during fall including all fish farms.
(TIFF)
