# Correction: Evaluation of a national operational salmon lice monitoring system—From physics to fish

**DOI:** 10.1371/journal.pone.0209949

**Published:** 2018-12-26

**Authors:** Mari Skuggedal Myksvoll, Anne Dagrun Sandvik, Jon Albretsen, Lars Asplin, Ingrid Askeland Johnsen, Ørjan Karlsen, Nils Melsom Kristensen, Arne Melsom, Jofrid Skardhamar, Bjørn Ådlandsvik

The Data Availability statement for this paper is incorrect. The correct statement is: The NorKyst800 model results are publicly available at http://thredds.met.no/thredds/catalog/fou-hi/norkyst800m/catalog.html and the salmon lice particle model results are available at http://www.imr.no/forskningsdata/smittepress_lakselus/. The underlying data for Figure 4 is available at http://metadata.nmdc.no/metadata-api/landingpage/362a7f635a2ea6d9d4b90fa1d1524565.
