# Supplementary figures and images for: Evaluation of a national operational salmon lice monitoring system—From physics to fish
Source: PLoS One. 2018 Jul 31;13(7):e0201338. doi: 10.1371/journal.pone.0201338 (PMC6067748; doi:10.1371/journal.pone.0201338)

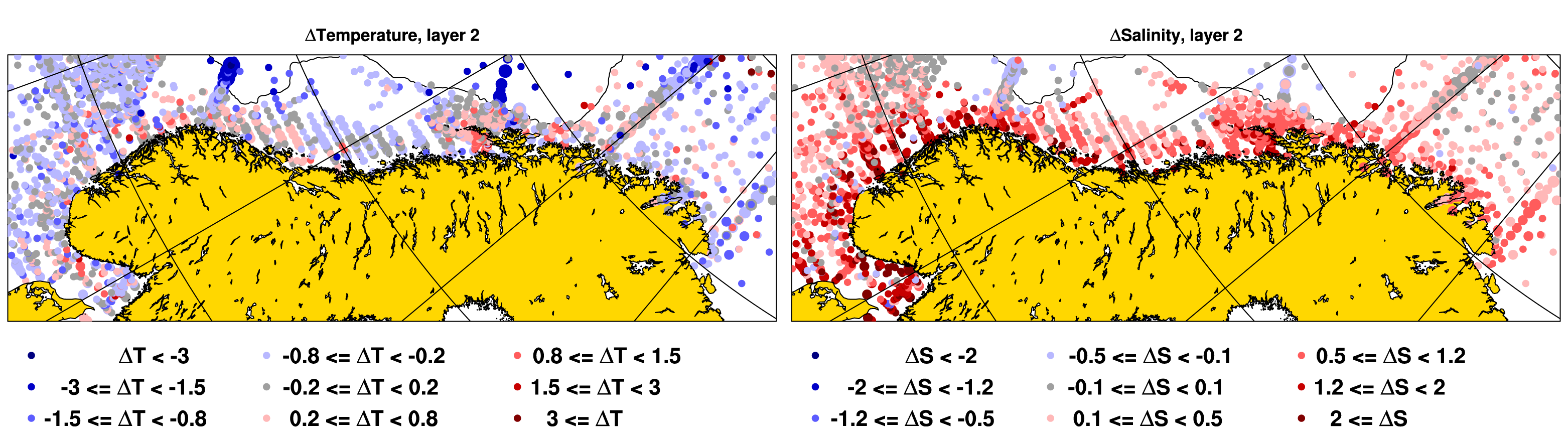

Supplement: S1 Fig — Each observation was assigned to the nearest grid cell in the model domain. The sizes of the dots are scaled so that their areas correspond to the number of observations in each grid cell. The dot size in the legend corresponds to the area when one observation is available. The magnitude of the model biases is given by the color coding which is defined in the graphic legend below the panels. The 1000 m depth isobaths are indicated by a black line. (TIF) [file pone.0201338.s002.tif]

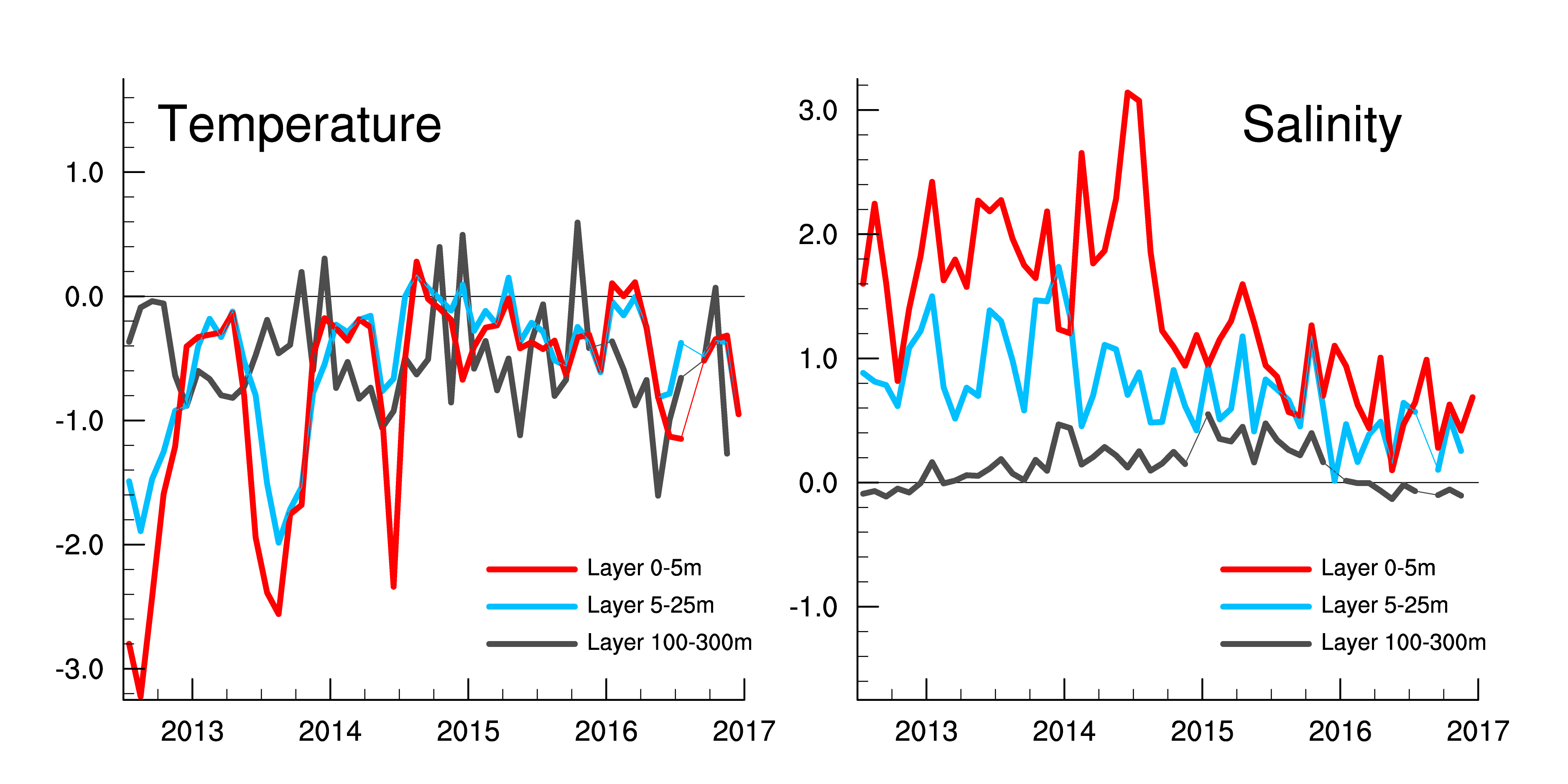

Supplement: S2 Fig — Time series of differences between model results and observations for temperature (left) and salinity (right). Differences are positive when model values are higher than the corresponding observations. Displayed here are monthly means of the layer average differences at the positions from which observations are available. Results are displayed for the three selected z layers as indicated by the line legends, corresponding to layers 1, 2 and 4 in Table 1. Thin lines bridge months with no data. (TIF) [file pone.0201338.s003.tif]

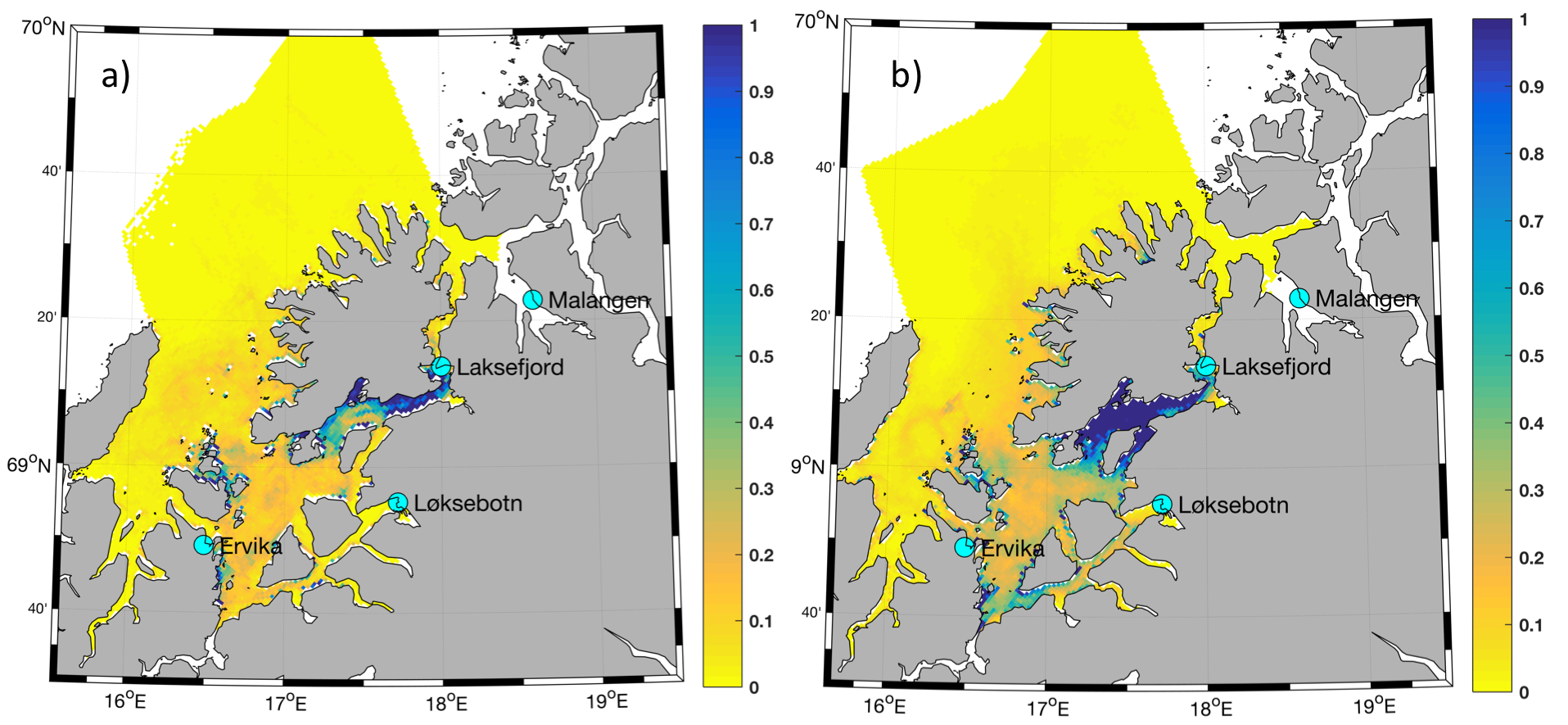

Supplement: S3 Fig — a) Operational model system, map used to select fishing location, b) Rerun of model system during fall including all fish farms. (TIFF) [file pone.0201338.s004.tiff]
